# Pharmacokinetic modelling to estimate intracellular favipiravir ribofuranosyl-5′-triphosphate exposure to support posology for SARS-CoV-2

**DOI:** 10.1093/jac/dkab135

**Published:** 2021-06-02

**Authors:** Henry Pertinez, Rajith K R Rajoli, Saye H Khoo, Andrew Owen

**Affiliations:** 1 Department of Pharmacology and Therapeutics, Materials Innovation Factory, University of Liverpool, Liverpool L7 3NY, UK; 2 Centre of Excellence in Long-acting Therapeutics (CELT), University of Liverpool, Liverpool L69 3BX, UK

## Abstract

**Objectives:**

Favipiravir has discrepant activity against SARS-CoV-2 *in vitro*, concerns about teratogenicity and pill burden, and an unknown optimal dose. This analysis used available data to simulate the intracellular pharmacokinetics of the favipiravir active metabolite [favipiravir ribofuranosyl-5′-triphosphate (FAVI-RTP)].

**Methods:**

Published *in vitro* data for intracellular production and elimination of FAVI-RTP in Madin–Darby canine kidney cells were fitted with a mathematical model describing the time course of intracellular FAVI-RTP as a function of favipiravir concentration. Parameter estimates were then combined with a published population pharmacokinetic model in Chinese patients to predict human intracellular FAVI-RTP. *In vitro* FAVI-RTP data were adequately described as a function of concentrations with an empirical model, noting simplification and consolidation of various processes and several assumptions.

**Results:**

Parameter estimates from fittings to *in vitro* data predict a flatter dynamic range of peak to trough for intracellular FAVI-RTP (peak to trough ratio of ∼1 to 1) when driven by a predicted free plasma concentration profile, compared with the plasma profile of parent favipiravir (ratio of ∼2 to 1). This approach has important assumptions, but indicates that, despite rapid clearance of the parent from plasma, sufficient intracellular FAVI-RTP may be maintained across the dosing interval because of its long intracellular half-life.

**Conclusions:**

Population mean intracellular FAVI-RTP concentrations are estimated to be maintained above the Km for the SARS-CoV-2 polymerase for 9 days with a 1200 mg twice-daily regimen (following a 1600 mg twice-daily loading dose on day 1). Further evaluation of favipiravir as part of antiviral combinations for SARS-CoV-2 is warranted.

## Introduction

The urgent global public health threat posed by COVID-19 has led the global scientific community to rigorously explore opportunities for repurposing existing medicines based upon either demonstration of auspicious antiviral activity against SARS-CoV-2 or a plausible mechanistic basis for anti-inflammatory/immunomodulatory activity. If sufficiently potent antiviral agents can be identified, there is potentially significant opportunity for deployment either as prophylaxis or in early infection to prevent development of severe disease. The role of antivirals in later stages of COVID-19 is less clear, but cannot be rigorously assessed until sufficiently potent antiviral drug combinations become available. Several groups have highlighted the importance of considering the fundamental principles of clinical pharmacology when selecting candidates for investigation as antiviral agents,^1–3^ including a nuanced understanding of the principles of plasma protein binding.^4^ For most successful antiviral drugs developed to date, a key principle is that plasma drug concentrations be maintained above *in vitro*-defined target concentrations (EC_90_ or protein binding-adjusted EC_90_ where available) for the duration of the dosing interval. It should be noted that neither the pharmacokinetic-pharmacodynamic relationship nor the pharmacokinetic parameters that best serve as surrogates for efficacy are yet understood for any SARS-CoV-2 antiviral drug. However, where the drug target resides intracellularly, and generation of an intracellular active metabolite is a prerequisite to unmask the pharmacophore, a more thorough understanding of the intracellular pharmacokinetics is required to rationalize doses required to maintain antiviral activity. For example, nucleoside-based drugs or prodrugs require intracellular phosphorylation by a cascade of host proteins, to generate tri-phosphorylated metabolites that exert the activity on the viral polymerase.^5^^,^^6^ Indeed, antiviral nucleoside analogues for HIV (e.g. tenofovir alafenamide), HCV (sofosbuvir) and several of the repurposing opportunities for SARS-CoV-2 (e.g. remdesivir) have active triphosphate metabolites with intracellular half-lives much longer than the parent drug in plasma.^7–9^ Therefore, for many nucleosides being explored for SARS-CoV-2 (including favipiravir and molnupiravir), the activity may be maintained across the dosing interval, despite plasma concentrations of the parent dropping below the EC_90_ at trough concentration (*C*_trough_).

Favipiravir is approved for influenza in Japan, but not elsewhere, and has been intensively studied as a potential antiviral intervention for several other RNA viruses; most recently SARS-CoV-2. For several reasons, considerable uncertainty exists about the suitability of favipiravir as a COVID-19 intervention. Concerns about teratogenicity and high pill burden may limit widespread uptake of the drug during early infection, particularly in the absence of concomitant contraceptive use in women of child-bearing age.^10^ Furthermore, *in vitro* studies of favipiravir in Vero-E6 cells infected with SARS-CoV-2 have yielded inconsistent findings^11–14^ and low potency (EC_90_ = 159 mM; 24.9 µg/mL) has been described in those studies that have shown activity.^14^ By comparison the EC_50_ of favipiravir against influenza A has an approximate range of 0.03 to 0.94 µg/mL.^10^^,^^15^ Favipiravir plasma concentrations also appear to diminish, the longer that patients receive the medicine,^10^ and studies in severe COVID-19 disease have shown that plasma exposures are almost entirely abolished.^16^ Despite the uncertainty and potential limitations, favipiravir has been demonstrated to exert antiviral activity against SARS-CoV-2 in the Syrian Golden Hamster model^17^ and, despite extremely high doses (1000 mg/kg intraperitoneally), *C*_trough_ values in this model were similar to those achieved in human studies of influenza (4.4 µg/mL in hamsters versus 3.8 µg/mL day 10 trough in humans).^17^^,^^18^ Cell-free models have also demonstrated the ability of the intracellular metabolite favipiravir ribofuranosyl-5′-triphosphate (FAVI-RTP) to directly inhibit the SARS-CoV-2 polymerase.^19^

The purpose of this work was to model, from published plasma pharmacokinetic profiles, the likely concentrations of the intracellular active moiety (FAVI-RTP) and evaluate whether putative target concentrations necessary to inhibit SARS-CoV-2 can be pharmacologically attained in humans.

## Methods

### Prior in vitro data

Data for the intracellular formation and catabolism of intracellular FAVI-RTP in Madin–Darby canine kidney (MDCK) cells following incubation with parent favipiravir were digitized from previously published work of Smee *et al*.^20^*In vitro* phosphorylation of favipiravir has been assessed in various cell lines^21^ and the production of FAVI-RTP in MDCK cells shown by Smee *et al*.^20^ is of similar magnitude to that shown in HeLa and Vero cells.^21^ The data of Smee *et al*.,^20^ however, characterize a full time course of the production and elimination of the FAVI-RTP metabolite rendering it the most suitable for this pharmacokinetic modelling exercise.

Briefly, the *in vitro* experiments carried out by Smee *et al*.^20^ involved incubation of confluent layers of MDCK cells in T-25 flasks with media containing favipiravir at 32, 100, 320 or 1000 µM for varying durations. Smee *et al*.^20^ included 10% FBS in media and plasma protein binding of favipiravir is relatively low in humans (54%).^10^ Therefore, given that the protein concentration in the medium was low in these incubations, it was assumed that the nominal *in vitro* media concentrations of favipiravir for the incubations equated to the free drug concentrations.

At given timepoints, medium was removed, cells were washed and lysed, and FAVI-RTP was quantified in the lysate using HPLC with UV detection. For catabolism/elimination experiments, MDCK cells were incubated with media containing favipiravir at the specified concentrations for 24 h to allow production and accumulation of FAVI-RTP, before medium was removed and replaced with favipiravir-free medium. Incubation then continued for a series of timepoints; at these timepoints medium was removed and cell lysate was assayed for FAVI-RTP. Smee *et al*.^20^ present their FAVI-RTP quantification in normalized units of pmol/10^6^ cells according to the cell counts of the incubations, which was taken to represent the normalized intracellular *amount* of FAVI-RTP. In this work, a value provided by Bitterman and Goss^22^ for the volume of an MDCK cell of 2.08 pL was then used to convert the FAVI-RTP quantification of Smee *et al*.^20^ into units of pmol/(10^6^ pL)  = pmol/µL = µM.

### Modelling of in vitro data

The data for intracellular production and elimination of FAVI-RTP were fitted with a mathematical model in the R programming environment (v 4.0.3)^23^ to describe and parameterize the observed data as a function of the incubation media concentrations and time. This fitting made use of the Pracma library^24^ and lsqnonlin function for non-linear regression.

**Figure 1.**
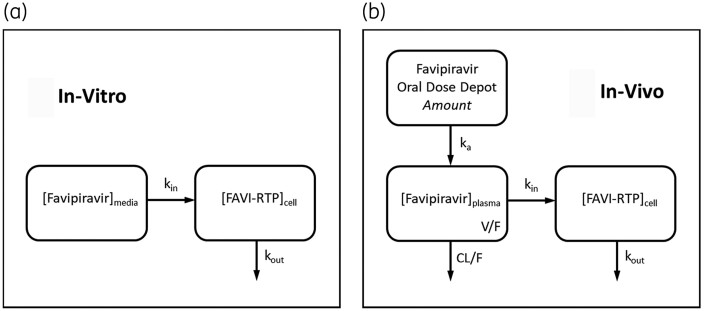
Schematics of mathematical models for favipiravir and intracellular FAVI-RTP kinetics as applied to *in vitro* data and used for *in vivo* simulations. [] denotes concentration.

Data for intracellular production (in the presence of favipiravir-containing media) and elimination of FAVI-RTP (on removal of favipiravir-containing media after a 24 h incubation and replacement with blank media) were combined into single time courses for each medium concentration, to then be described with the following ordinary differential (rate) equation mathematical model (schematic provided in Figure 1a):

$$\text{Equation 1: }\frac{d{\left[\text{FAVI}-\text{RTP}\right]}_{\text{cell}}}{dt}={\;k}_{\text{in}}\times {\left[\text{favipiravir}\right]}_{\text{media}}-k_{\text{out}}\times {\left[\text{FAVI}-\text{RTP}\right]}_{\text{cell}}$$

where [] denotes concentration. The initial condition for [FAVI-RTP]_cell_ is set to zero at time zero and [favipiravir]_media_ switched to equal zero at the 24 h timepoint to end the production rate of FAVI-RTP and allow only the elimination rate to describe observed, declining concentrations from that time forward. The parameter k_in_ has units of time^−1^ and simultaneously describes net influx/diffusion of parent favipiravir from media into the cell and its (net) subsequent conversion into FAVI-RTP. The use of k_in_ therefore simplifies a more detailed description of favipiravir net influx into cells and conversion into FAVI-RTP into an empirical first-order process dependent on media concentration of favipiravir, to enable use of the information in the available data, where parent favipiravir itself is not quantified.

Smee *et al*.^20^ do not explicitly quote the volume of media used in their incubations, but given the T-25 flasks used, a media volume of 5–10 mL could be expected. Across the lowest to highest favipiravir media concentrations in the data (32 and 1000 µM), this therefore translates to a range of 0.16 to 10 µmol favipiravir present in the incubations at time = 0. The maximum amount of intracellular FAVI-RTP produced after 24 h in the 1000 µM incubation was 332 pmol/10^6^ cells; with incubations declared to contain approximately 7 × 10^6^ cells on average, this gives a maximum total amount of 2324 pmol = 2.3 nmol of FAVI-RTP converted from favipiravir in 1:1 stoichiometry, which is ≪1% conversion of the total amount of favipiravir available in the 1000 µM incubation at the start (with similar calculations demonstrable at the other media concentrations). Therefore the [favipiravir]_media_ term in Equation 1 can be considered approximately constant for a given time course dataset of incubations at a specified favipiravir concentration. In turn, this renders Equation 1 equivalent to a zero-order constant input model with first-order elimination and with the zero-order input being switched off at 24 h when media containing favipiravir was replaced with blank media.

### In vivo intracellular simulations

The model and parameter estimates from the fitting to *in vitro* intracellular data, describing intracellular FAVI-RTP concentrations as a function of the media incubation concentrations, were then taken forward and combined with a population pharmacokinetic model for plasma exposure for favipiravir described by Wang *et al*.^18^ in a Chinese population receiving the drug for influenza, substituting the media incubation concentration driving the intracellular FAVI-RTP production rate with the free plasma concentration predicted by the population pharmacokinetic model. This provided a prospective simulation of *in vivo* intracellular concentrations of FAVI-RTP (assuming cells of similar disposition to MDCK cells) as a function of *in vivo* plasma exposure.

The population pharmacokinetic model of Wang *et al*.^18^ is a one-compartment pharmacokinetic disposition model with first-order absorption. Therefore, the equations for the model for *in vivo* intracellular simulations were as follows (schematic provided in Figure 1b):

$$\text{Equation 2: }\frac{d{\;A}_{\text{Favi}\_\text{depot}}}{dt}=-k_{a}\times {\;A}_{\text{Favi}\_\text{depot}}$$

$$\text{Equation 3: }\frac{d{\left[\text{favipiravir}\right]}_{\text{plasma}}}{dt}=\left(k_{a}\times {\;A}_{\text{Favi}\_\text{depot }}–\;\text{CL}\times {\left[\text{favipiravir}\right]}_{\text{plasma}}\right)/V$$

$$\text{Equation 4: }\frac{d{\left[\text{FAVI}-\text{RTP}\right]}_{\text{cell}}}{dt}=\left(k_{\text{in}}\times {\left[\text{favipiravir}\right]}_{\text{plasma}}\times {\;\text{Fu}}_{\text{plasma}}\right)-{\;k}_{\text{out}}\times {\left[\text{FAVI}-\text{RTP}\right]}_{\text{cell}}$$

where [] denotes concentration, CL and V are apparent values CL/F and V/F, and k_in_ and k_out_ used values derived from the *in vitro* model fitting. The Wang *et al*.^18^ model also incorporated a time-dependent effect on CL, representative of favipiravir autoinduction of its own elimination, where:

$$\text{Equation 5:CL}={\;\text{CL}}_{\text{day}\_0}\times \left(1+0.0614\times \text{days}\;\text{of}\;\text{dosing}\right)$$

The model assumes a minimal proportion of the total mass balance of favipiravir transfers in from the plasma before conversion into the intracellular FAVI-RTP (similar to how the *in vitro* model assumes a constant [favipiravir]_media_) and was therefore similar in some respects to a pharmacokinetic-pharmacodynamic effect compartment model.

Pharmacokinetic parameter population inter-individual variabilities estimated by Wang *et al*.^18^ were used to simulate a population of 1000 sets of pharmacokinetic parameters and their resultant predicted [favipiravir]_plasma_ and [FAVI-RTP]_cell_ profiles, from which 90% prediction interval profile envelopes were calculated. No population distribution of body weight was incorporated into this simulation, which is equivalent therefore to assuming each simulated subject had a body weight of 70 kg according to the Wang *et al*.^18^ population pharmacokinetic model. No inter-individual variability in the k_in_ or k_out_ parameters was available or assumed. Parameter values used, and their inter-individual variabilities, quoted from the population pharmacokinetic model of Wang *et al*.^18^ are provided in Table 1, with Fu_plasma_ set at 0.46.^10^

**Table 1.** Summary of favipiravir population pharmacokinetic model parameter estimates used for simulation, from Wang *et al*.^18^

Parameter	Est.	Inter-individual variance (*ω*^2^, log parameters)
F (apparent, fractional)	1 (fixed)	0.0921
k_a_ (h^−1^)	1.5	1.05
CL/F (L/h)	2.96	0.274
V/F (L)	37.1	0.128
Time-dependent effect on CL/F (% per day)	6.14	–
Inter-individual covariance for k_a_ and V/F (*ω*^2^_ka_∼*ω*^2^_V/F_)	–	0.23

## Results

### Modelling of in vitro data

**Figure 2.**
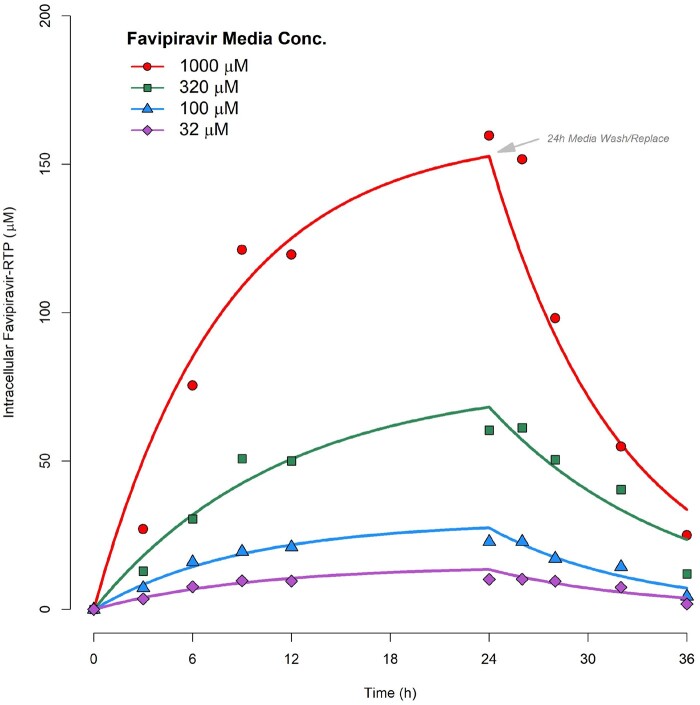
Pharmacokinetic model fittings to *in vitro* time courses of [FAVI-RTP]_intracellular_ generated by Smee *et al*.^20^ in MDCK monolayers.

**Figure 3.**
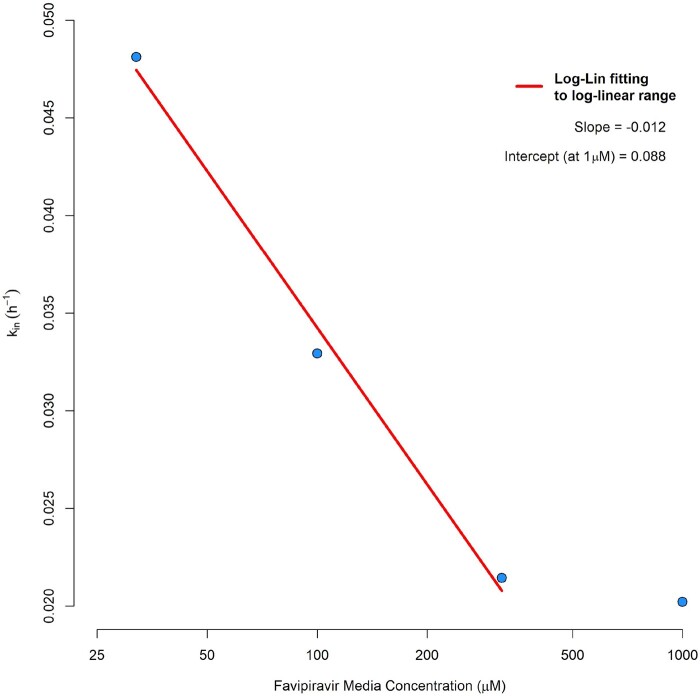
Log-linear fitting to k_in_ as a function of media favipiravir concentration.

Data from the observed [FAVI-RTP]_cell_ from Smee *et al*.^20^ at the four media concentrations investigated, overlaid with the model fittings, are given in Figure 2, with parameter estimates and associated % relative standard errors in Table 2. The model was deemed to provide a satisfactory description of the observed data with acceptable precision of parameter estimates. However, it should be noted that k_in_ values were not observed to be constant across the media incubation concentrations. A plot of k_in_ versus [favipiravir]_media_ (the latter on a log scale) is provided in Figure 3, indicating that some form of saturable relationship is most likely present, which may require an E_max_-type model to be accurately described over the full range of media concentrations. However, with data available at only four media concentrations there was insufficient information to adequately fit such a saturable model. A log-linear model has therefore been fitted instead to the log-linear portion of the k_in_ versus [favipiravir]_media_ curve ([favipiravir]_media_ ranges from 32 to 320 µM, which also encompasses a typical range of *in vivo* plasma concentrations under standard human dosing regimens) and is overlaid in Figure 3, where:

$$\text{Equation 6:}k_{\text{in}}=\text{ln}\left({\left[\text{favipiravir}\right]}_{\text{media}}\right)\times {\;k}_{\text{in}\_\text{slope}}+{\;k}_{\text{in}\_\text{intercept}}$$

**Table 2.** Parameter estimates from *in vitro* pharmacokinetic model fittings

[Favipiravir]_media_	k_in_ (h^−1^)	k_out_ (h^−1^)
1000 µM		
est.	0.020	0.126
%RSE	13.9	11.4
320 µM		
est.	0.021	0.089
%RSE	14.7	15.6
100 µM		
est.	0.033	0.112
%RSE	12.0	10.7
32 µM		
est.	0.048	0.105
%RSE	15.3	14.3

%RSE, % relative standard error.

The values of k_in_slope_ and k_in_intercept_ were then taken forward to the *in vivo* simulations instead of a mean value of k_in_, or the value from one [favipiravir]_media_ fitting alone, and were used via Equation 6 during simulations to calculate the value of k_in_ required for any free plasma concentration at any given time as an input parameter value for Equation 4. k_out_ estimates from fittings were consistent at the four *in vitro* [favipiravir]_media_ concentrations. Therefore, the mean of the four estimates (0.108 h^−1^) was taken as the input value for simulations using Equation 4.

### In vivo intracellular simulations

**Figure 4.**
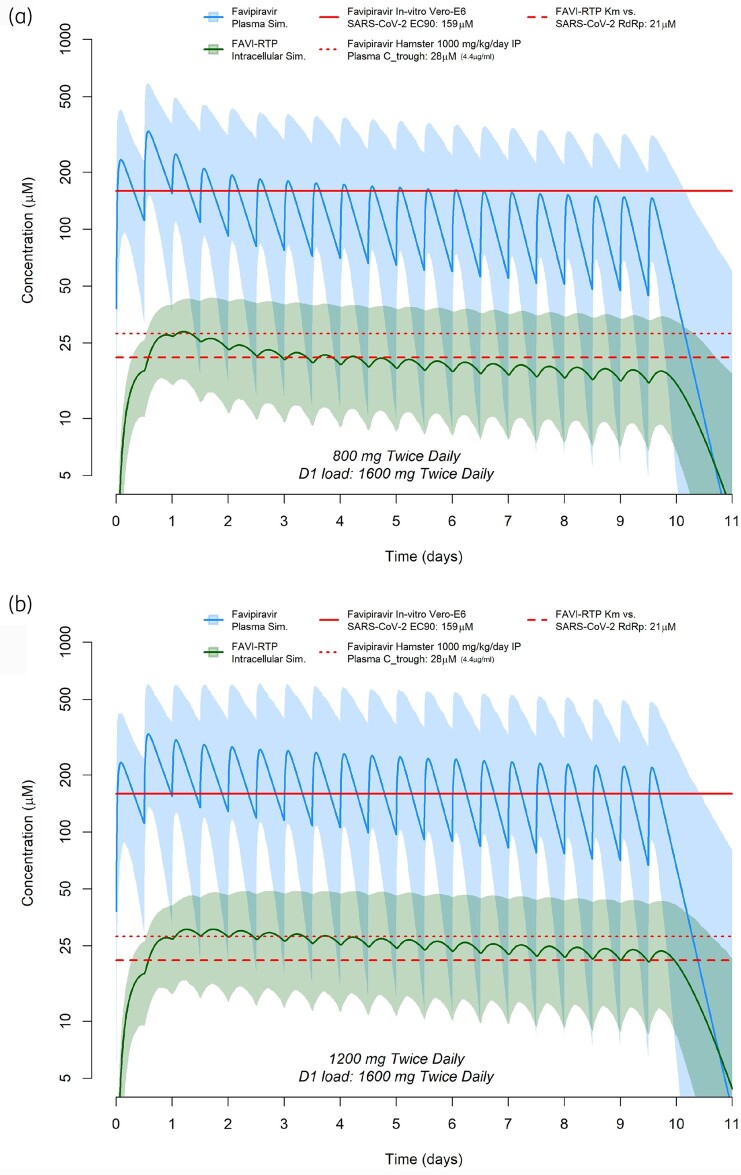
Favipiravir plasma and intracellular FAVI-RTP concentration predictions based on the population pharmacokinetic model of Wang *et al*.^18^ combined with *in vitro* intracellular pharmacokinetic modelling, for a dosing regimen of 1600 mg twice daily as a loading dose on day 1 followed by 800 mg twice-daily maintenance dosing for 9 further days (a) or 1600 mg twice daily as a loading dose on day 1 followed by 1200 mg twice-daily maintenance dosing for 9 further days (b). The dashed red line represents the previously published Km for inhibition of SARS-CoV-2 polymerase by FAVI-RTP, ^19^ the dotted red line represents *C*_trough_ plasma concentrations of favipiravir following a 1000 mg/kg/day dose in hamsters^17^ and the solid red line represents the *in vitro* EC_90_ of favipiravir against SARS-CoV-2 in Vero-E6 cells.^14^

Simulations of predicted *in vivo* plasma and intracellular exposures for favipiravir and FAVI-RTP are shown in Figure 4(a) for a dosing regimen of 1600 mg twice daily as a loading dose on day 1 followed by 800 mg twice-daily maintenance dosing for 9 further days. Simulations of predicted *in vivo* plasma and intracellular exposures for favipiravir and FAVI-RTP are shown in Figure 4(b) for a dosing regimen of 1600 mg twice daily as a loading dose on day 1 followed by 1200 mg twice-daily maintenance dosing for 9 further days. In both cases, reference to the Km (Michaelis–Menten constant) for FAVI-RTP against the SARS-CoV-2 RNA-dependent RNA polymerase (RdRp) enzyme^19^ is overlaid. The plasma target exposure based on the *in vitro* EC_90_ of favipiravir of 159 µM against SARS-CoV-2^14^ is also shown, but should be interpreted with caution owing to the lack of clarity in *in vitro* free drug concentrations and whether human plasma binding is high or low affinity.

## Discussion

While studies in the Syrian Golden Hamster have demonstrated the effectiveness of favipiravir against SARS-CoV-2, *in vitro* activity data generated in the Vero-E6 cell model have questioned the utility of the molecule when the derived target concentrations are compared with the pharmacokinetics after administration to humans. Ultimately, robustly designed and executed clinical trials will be required to determine the utility of favipiravir for SARS-CoV-2, but understanding the mechanisms that underpin the clinical pharmacology is important to understand the plausibility for evaluation, which should underpin the selection of candidates for clinical evaluation. Furthermore, an *a priori* understanding of likelihood of success for future candidates can only evolve from a thorough understanding of the pharmacokinetic-pharmacodynamic rules of engagement for SARS-CoV-2, which currently do not exist. Antiviral drugs have only thus far been unequivocally successful for other viruses when given in combination and the requirement of combinations to improve potency and/or stem emergence of resistance requires careful consideration so as not to obviate the lessons that should be learned from other pathogens. Notwithstanding, favipiravir has been evaluated at several different doses and schedules in numerous clinical trials globally, with mixed outcomes.^25–27^ As of 29 December 2020, a total of 44 trials were listed on clinicaltrials.gov aiming to evaluate favipiravir, predominantly as a monotherapy (with some exceptions) and in various-use cases.

The current analysis aimed to apply a pharmacokinetic modelling approach to better understand the potential efficacy of favipiravir for SARS-CoV-2 at doses readily achievable in humans. The simulations synthesized available data for intracellular kinetics of FAVI-RTP in MDCK cells, plasma pharmacokinetics in a Chinese patient population, *in vitro*-derived antiviral activity data (EC_90_) and cell-free inhibition data for FAVI-RTP against the SARS-CoV-2 RNA polymerase. Importantly, this modelling approach indicates that, despite rapid clearance of the parent drug from plasma, the peak to trough variability in intracellular FAVI-RTP is such that activity may be maintained across the dosing interval because of the long intracellular half-life. The simulations indicate that the population mean intracellular FAVI-RTP concentrations will be maintained above the FAVI-RTP Km for the SARS-CoV-2 polymerase for 3 days during an 800 mg twice-daily dosing regimen and 9 days during a 1200 mg twice-daily dosing regimen (both regimens including a 1600 mg twice-daily loading dose on day 1). The ratio of peak to trough concentrations in plasma on the second day of dosing for both 800 and 1200 mg maintenance dosing regimens is approximately 2.3 to 1, while for intracellular FAVI-RTP the peak to trough ratio is a flatter 1.1 to 1. Importantly, the flatter intracellular pharmacokinetic profile of the phosphorylated form of favipiravir is in keeping with observations for other antiviral nucleoside/nucleotide analogues such as tenofovir-diphosphate,^28^^,^^29^ which underpins the efficacy of these drugs for other viruses.

The current approach has several important limitations that should be recognized. Favipiravir pharmacokinetic exposures have been demonstrated to be lower in American and African patients than in Chinese patients^30^ and so the simulations may not be widely applicable across different ethnicities. This potential inter-ethnic difference in favipiravir exposure may reflect a wider variability in favipiravir pharmacokinetics that may become better characterized as further trials with it are carried out. Simulations here should be considered in the specific context therefore of the Wang *et al*.^18^ population pharmacokinetic dataset and model that informed the favipiravir pharmacokinetic parameters for this exercise.

The modelling approach applied a direct *in vitro* to *in vivo* extrapolation of k_in_ and this should be considered as a major assumption as it directly presumes the *in vitro* favipiravir_media_ concentration is representative of the free plasma concentration as derived from the pharmacokinetic model and that the umbrella k_in_ parameter, which consolidates various underlying uptake and conversion processes, is directly translatable. Importantly, the presented intracellular predictions are specific to data generated on intracellular kinetics in MDCK cells. Therefore, the accuracy of the intracellular FAVI-RTP concentrations will be dependent upon the similarity of relevant human *in vivo* cells in terms of the *in vitro* uptake/elimination as well as the rate and extent of metabolic activation of favipiravir to its triphosphorylated active form. Although *in vivo* intracellular phosphorylation of favipiravir is yet to be well characterized in the literature, favipiravir intracellular phosphorylation to FAVI-RTP of a similar magnitude to that seen by Smee *et al*.^20^ in MDCK cells has also been demonstrated *in vitro* in other cell lines, including the Vero cell line.^21^ Given that the correlation between *in vitro* and *in vivo* intracellular phosphorylation has been demonstrated in Vero cells for various other NRTIs^31^ and that favipiravir *in vitro* phosphorylation is similar in MDCK and Vero cells, by extension, it remains reasonable that *in vitro* phosphorylation in MDCK cells remains an indicator of intracellular phosphorylation *in vivo*. It would be a reasonable avenue for future work to investigate validation of the *in vitro* intracellular modelling and extrapolation approach applied here with other nucleoside analogues where suitable intracellular *in vitro* and *in vivo* data both exist.

Intracellular predictions are limited further in this work in that there are no data with which to model the inter-patient variability in the intracellular uptake or conversion into FAVI-RTP and so intracellular concentration variability shown in Figure 3 is only derived from intracellular variability in plasma exposure. Finally, the intracellular prediction is driven by the estimated free plasma concentration, whereas *in vivo* it is possible local tissue free drug concentrations at the target organ for which there are no available data may be higher or lower than in plasma.

Despite these limitations, additional confidence in the predictions comes from two important sources. Firstly, following the first day of dosing, there is generally good agreement between the point at which the plasma favipiravir concentrations intersect the *in vitro*-derived EC_90_ and the corresponding intracellular FAVI-RTP value being close to its Km value derived separately in a cell-free system with the SARS-CoV-2 polymerase (Figure 3). It should be noted that no data are available with which to derive a protein-adjusted EC_90_ value of favipiravir. Secondly, the clear activity of favipiravir in the Syrian Golden Hamster model when *C*_trough_ values are similar to those in humans gives additional confidence in the predictions.^17^

In summary, these simulations indicate that favipiravir maintenance doses between 800 and 1200 mg twice daily may be sufficient to provide therapeutic concentrations of the intracellular FAVI-RTP metabolite across the dosing interval. Further evaluation of favipiravir as an antiviral for SARS-CoV-2 appears to be warranted and will provide additional clarity on its putative utility. However, the recent emergence of variants of the virus requires careful consideration of the drug resistance threat posed by using repurposed agents as monotherapies, particularly when they are likely not to be fully active in all patients. The polymerase, along with the protease and spike, are likely to be extremely important drug targets for new chemical entities and care should be taken not to compromise their utility before the first-generation specific SARS-CoV-2 antivirals emerge.
